# Using Targeted Metabolomics to Unravel Phenolic Metabolites of Plant Origin in Animal Milk

**DOI:** 10.3390/ijms25084536

**Published:** 2024-04-20

**Authors:** Vicente Agulló, Claudia Favari, Niccolò Pilla, Letizia Bresciani, Francisco A. Tomás-Barberán, Alan Crozier, Daniele Del Rio, Pedro Mena

**Affiliations:** 1 Human Nutrition Unit, Department of Food & Drug, University of Parma, 43125 Parma, Italy; claudia.favari@unipr.it (C.F.); niccolo.pilla@studenti.unipr.it (N.P.); letizia.bresciani@unipr.it (L.B.); daniele.delrio@unipr.it (D.D.R.); 2 Quality, Safety, and Bioactivity of Plant Foods Research Group, Laboratory of Food & Health, CEBAS–CSIC, Espinardo P.O. Box 164, 30100 Murcia, Spain; fatomas@cebas.csic.es; 3 Department of Chemistry, King Saud University, Riyadh 11451, Saudi Arabia; alan.crozier44@gmail.com; 4 School of Medicine, Dentistry and Nursing, University of Glasgow, Glasgow G12 8QQ, UK; 5 Microbiome Research Hub, University of Parma, 43125 Parma, Italy

**Keywords:** (poly)phenol, cow, goat, UHPLC-QqQ, inter-individual variability, dietary supplementation

## Abstract

Milk holds a high nutritional value and is associated with diverse health benefits. The understanding of its composition of (poly)phenolic metabolites is limited, which necessitates a comprehensive evaluation of the subject. This study aimed at analyzing the (poly)phenolic profile of commercial milk samples from cows and goats and investigating their sterilization treatments, fat content, and lactose content. Fingerprinting of phenolic metabolites was achieved by using ultra-high-performance liquid chromatography coupled with triple-quadrupole mass spectrometry (UHPLC-QqQ-MS/MS). Two hundred and three potential microbial and phase II metabolites of the main dietary (poly)phenols were targeted. Twenty-five metabolites were identified, revealing a diverse array of phenolic metabolites in milk, including isoflavones and their microbial catabolites equol and *O*-desmethylangolensin, phenyl-γ-valerolactones (flavan-3-ol microbial catabolites), enterolignans, urolithins (ellagitannin microbial catabolites), benzene diols, and hippuric acid derivates. Goat’s milk contained higher concentrations of these metabolites than cow’s milk, while the sterilization process and milk composition (fat and lactose content) had minimal impact on the metabolite profiles. Thus, the consumption of goat’s milk might serve as a potential means to supplement bioactive phenolic metabolites, especially in individuals with limited production capacity. However, further research is needed to elucidate the potential health effects of milk-derived phenolics.

## 1. Introduction

Milk is secreted by female mammals to provide the energy and nutrients necessary for the growth and development of newborns during the first months of life. In terms of composition, it consists of water, lipids, carbohydrates (both lactose and oligosaccharides), high-biological-value proteins, vitamins (mainly from the B complex), minerals such as calcium, and a variety of cells and other bioactive substances [1,2]. The nutritional richness of milk, as a significant source of essential macro- and micronutrients, provides various health benefits also for adult humans. For instance, in relation to cardiovascular disease prevention, calcium is involved in blood pressure regulation, and some casein-derived peptides exhibit antihypertensive effects [3,4]. Cow’s milk is also a good dietary source of calcium, and this may have a positive impact on bone mineral density [5,6]. But, despite these health-promoting benefits, the influence of milk consumption on human health is controversial due to its saturated fat content and an intolerance to lactose in some people. As a complex food matrix, the different components of milk may have beneficial or adverse effects in different (patho)physiological conditions. However, there is no strong evidence to support claims that moderate milk consumption should be excluded from the diet. Indeed, to the contrary, milk and its derivative products are included in recommendations for a healthy diet [2,5].

Milk composition is influenced by a number of factors, including genetics, breed, age, health status, lactation stage, and the animal’s diet. In this regard, the animal’s diet can be designed to modulate the composition and nutritional profile of milk [7]. Pasture feeding has been shown to have a positive impact on the nutrient profile of milk, increasing the content of certain nutrients, and meeting the needs of consumers who desire healthy and sustainable dairy products that are perceived to be “natural” [8,9,10]. Feeding regimens may also impact milk composition, affecting changes in lipids, peptides, and secondary metabolites, including those derived from (poly)phenols [1].

(Poly)phenols are a broad family of plant secondary compounds related to the health benefits attributed to fruit and vegetable consumption [11,12]. Once consumed, they are extensively metabolized by mammalian and microbial enzymes and can be present in biological fluids, including milk. Some studies have demonstrated that the phenolic metabolites in milk originate from the animal’s diet and following transformation by gut microorganisms. Isoflavones and lignans, as the predominant (poly)phenolic compounds in forage, and their microbial derivatives, as the result of ruminal fermentation, have been identified in cow’s milk [13,14,15,16,17,18]. However, the literature related to other phenolic metabolites that occur in milk is scarce, and an overall assessment of the phenolic composition of milk of various origins is lacking.

In this study, an ultra-high-performance liquid chromatography method coupled with triple-quadrupole mass spectrometry (UHPLC-QqQ-MS) facilitating the analysis of >200 phenolic metabolites was used to determine the (poly)phenolic profile of different commercial milk samples. The study used cow’s and goat’s milk and investigated the effects of thermal treatment (mild pasteurization, UHT, microfiltration), fat content (whole, semi-skimmed), and lactose content (normal, lactose-free). The information collected was used to estimate the (poly)phenol metabolite intake attributable to a glass of milk.

## 2. Results

### 2.1. Qualitative Analysis of Milk Samples

Thirteen different types of commercial milk were analyzed (Table 1).

The diversity of milk phenolic metabolites was evaluated by targeting 203 potential compounds belonging to the following phenolic classes and their microbial catabolites: isoflavones, flavan-3-ols, flavonols, hydroxycinnamic acids, hydroxybenzoic acids, hippuric acids, benzene diols (also known as catechols), lignans, and ellagitannins. Twenty-five (poly)phenolic metabolites were detected (Table 2). These belonged to various classes, including isoflavones, phenyl-γ-valerolactones (flavan-3-ol microbial catabolites), enterolignans, urolithins (ellagitannin microbial catabolites), hippuric acids, and benzene diols.

### 2.2. Quantitative Analysis of Milk Samples

#### 2.2.1. Isoflavone Metabolites

Isoflavones are phytoestrogens that are structurally similar to mammalian-synthesized estrogen, which have health-promoting properties. The quantification of isoflavone metabolites is presented in Table 3. Equol derivatives, colonic catabolites of the soy isoflavone daidzein, were the main isoflavone conjugates identified in the milk samples. They were also among the main phenolic compounds in milk. Goat’s milk had the highest concentration of isoflavone metabolites, with 3904 and 2350 ng/100 mL, for semi-skimmed (USG) and whole milk (UWG) samples, respectively. Respectively, these values were 6.4- and 3.9-fold higher than the highest value for the other types of milk.

**Figure 1:**
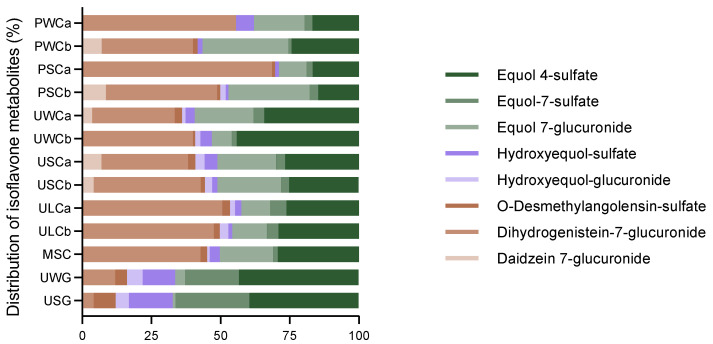
Relative concentration of isoflavone metabolites in the milk samples. Values are represented as a percentage of the total concentration.

Figure 1 illustrates the relative concentration of isoflavone metabolites in each type of milk, revealing notable differences between cow’s and goat’s milk. Dihydrogenistein-7-glucuronide was the most abundant metabolite in cow’s milk, followed by equol derivatives. In contrast, goat’s milk had equol derivatives as the most representative metabolites, while dihydrogenistein-7-glucuronide was present in considerably lower proportions compared with cow’s milk. Hydroxyequol derivatives were more prominent in goat’s milk compared with cow’s milk. These derivatives were relatively minor components in cow milk samples, accounting for ca. <7% of the total isoflavone metabolites.

#### 2.2.2. Flavan-3-ol Metabolites

Flavan-3-ols, also known as flavanols, are flavonoids that are found as monomers but also occur as oligomeric and polymeric structures called proanthocyanins or condensed tannins. Phenyl-γ-valerolactones, the main gut-microbiota-derived catabolites of flavan-3-ols, occurred mainly as phase II sulfate conjugates in all the milk samples. In contrast, no phase II derivatives of the monomeric (–)-epicatechin were detected (Table 4). Goat’s milk contained the highest concentrations of phenyl-γ-valerolactone derivatives; the semi-skimmed milk (USG) had a 10-fold higher level than the whole milk (UWG), and a 47-fold higher level than cow’s milk.

**Figure 2:**
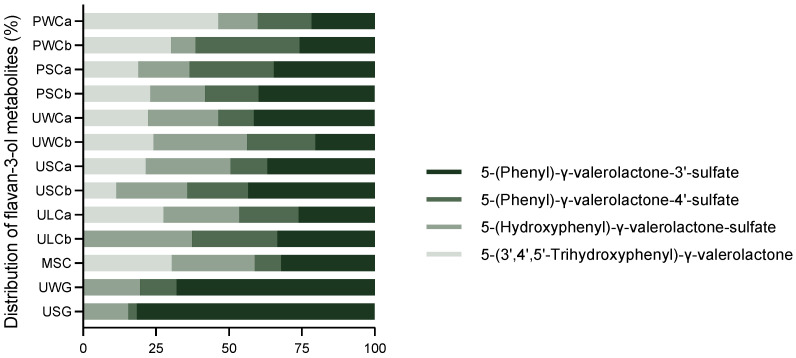
Relative concentration of flavan-3-ol metabolites in the milk samples. Values are represented as a percentage of the total concentration (%).

Figure 2 displays the relative concentrations of phenyl-γ-valerolactones in all the milk samples, showing heterogeneous results even within the same type of milk when considering two different brands. For most samples, 5-(phenyl)-γ-valerolactone-sulfates were the most abundant metabolites accounting for, for instance, approximately 80% of flavan-3-ols in goat’s milk. Interestingly, 5-(3′,4′,5′-trihydroxyphenyl)-γ-valerolactone, a catabolite derived from trihydroxylated flavan-3-ols like (epi)gallocatechin, was detected only in cow’s milk, except for one lactose-free UHT cow’s milk (ULCa and ULCb).

#### 2.2.3. Lignan Metabolites

Lignans are classified as phytoestrogens, which are catabolized by gut microbiota to the enterolignans enterodiol and enterolactone, which undergo phase II metabolism to form sulfate or glucuronide conjugates. Although both enterolignans were found in the milk samples, only enterolactone metabolites were quantified (Table 5), as the levels of enterodiol and enterodiol-sulfate were found to be below the limit of quantification in all the samples. In terms of metabolite concentration, the highest amounts were found in whole UHT cow’s milk (UWCa and UWCb) and lactose-free UHT cow’s milk (ULCa and ULCb), followed closely by semi-skimmed UHT goat’s milk (USG). Approximately 3-fold lower levels were detected in microfiltered, semi-skimmed cow’s milk (MSC), as well as in one brand of semi-skimmed UHT cow’s milk (USCb) and whole UHT goat’s milk (UWG).

**Figure 3:**
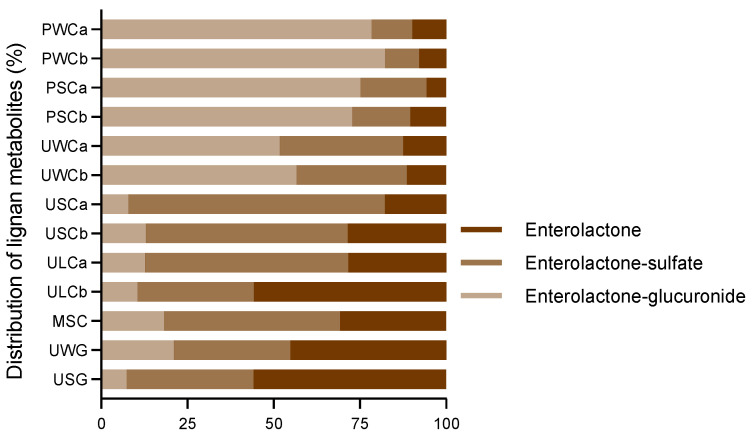
Relative concentration of lignan metabolites in the milk samples. Values are represented as a percentage of the total concentration.

The relative concentrations of enterolactone derivatives in all the milk samples are presented in Figure 3. Enterolactone-glucuronide was the most abundant enterolignan metabolite for whole (PWC) and semi-skimmed pasteurized cow’s milk (PSC) and whole UHT cow’s milk (UWC), representing 50–80% of the enterolignan profile. However, there were lower levels of this metabolite in semi-skimmed UHT (USC), lactose-free UHT (ULC), and microfiltered semi-skimmed cow’s milk (MSC), as well as whole (UWG) and semi-skimmed UHT goat’s milk (USG), where it accounted for only 7–20% of the lignan-derived components. In these latter milk samples, the most representative metabolites were enterolactone and enterolactone-sulfate, depending on the specific milk sample.

#### 2.2.4. Ellagitannin Metabolites

Ellagitannins are hydrolyzable tannins found in some berries, nuts, and pomegranates. Upon ingestion, they can be hydrolyzed by gut enzymes and the microbiota to release ellagic acid, which can undergo further microbial catabolism to form urolithins. Urolithins were detected in the milk samples (Table 6). The highest amounts were found in goat’s milk. The total concentration of urolithins in semi-skimmed UHT goat’s milk (USG) was 22-fold higher than in whole UHT goat’s milk (UWG) and approximately 300-fold higher than in the cow milk samples. The presence of urolithins in these milks indicates that there were sources of ellagitannins in the ingested forage and fodders.

**Figure 4:**
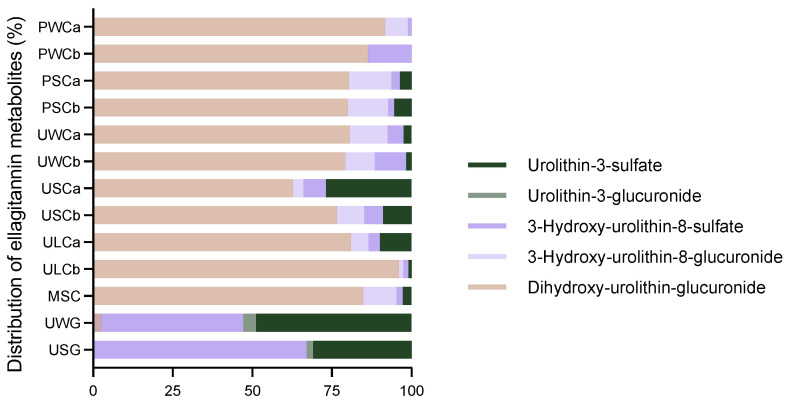
Relative concentration of ellagitannin metabolites in the milk samples. Values are represented as a percentage of the total concentration (%).

Figure 4 illustrates the relative concentration of the different urolithins in the milk samples. Dihydroxy-urolithin-glucuronide (also known as urolithin C-glucuronide) was the most abundant urolithin in cow’s milk, while 3-hydroxy-urolithin-8-sulfate (also known as urolithin A-sulfate) and urolithin-3-sulfate (also known as urolithin B-sulfate) were the most prevalent in goat milk samples. Of note, 3-hydroxy-urolithin-8-glucuronide (also known as urolithin A-glucuronide) was exclusive to cow milk samples and urolithin-3-glucuronide (also known as urolithin B-glucuronide) to goat’s milk.

#### 2.2.5. Hippuric Acids

Hippuric acid is a metabolite produced by the conjugation of benzoic acid with glycine in the liver. The presence of (poly)phenols in the diet has been shown to increase the production of hippuric acid. The quantification of hippuric acid and a hydroxylated derivative is presented in Table 7. The highest hippuric acid value was found in the semi-skimmed UHT goat’s milk (USG), followed by one of the two whole pasteurized cow milk samples (PWCa). The lowest values were observed in semi-skimmed pasteurized cow’s milk (PSC) and some whole (UWC) and semi-skimmed UHT (USC) cow milk samples, with values around 2-fold lower than the highest one. Regarding individual metabolites, hippuric acid was clearly the most abundant metabolite in all the samples, with 3ʹ-hydroxyhippuric acid representing only 1–6% of the total concentration of hippuric acid metabolites.

#### 2.2.6. Benzene Diol Metabolites

Benzene diols are produced through the catabolism of many (poly)phenolic compounds. The quantification of two hydroxybenzene sulfates is represented in Table 8. The highest values were found in goat’s milk, where the concentration of two isomers in semi-skimmed UHT goat’s milk (USG) was 3.6-fold higher than in whole UHT goat’s milk (UWG), and approximately 18-fold higher than in cow’s milk. The proportion of the two benzene-1/2-ol-sulfates ranged from 26% to 70% of isomer 1 and varied among samples, without a clear pattern among milk types.

## 3. Discussion

The profiling of the phenolic composition of commercial cow and goat milks, using a targeted approach, revealed the presence of a substantial number of phenolic metabolites. This is of interest as these are derived principally from plant-based (poly)phenols in animal foodstuffs, and following ingestion by cows and goats, they undergo metabolism, appearing in the circulatory system and being transported to the milk as well as being excreted in the urine. Consumption of milk can, therefore, be a useful supplement, adding to the amounts of these potentially protective compounds in humans that are derived principally from a (poly)phenol and aromatic amino acid-rich plant-based diet. The in vivo production of a number of colonic phenolic metabolites, such as equol, phenyl-γ-valerolactones, enterolignans, and urolithins are characterized by high inter-individual differences [20]. Intake from milk might, therefore, help to overcome or reduce this microbiota-related variability.

Isoflavones, including genistein, daidzein, glycitein, formononetin, and biochanin, are the predominant phenolic compounds found in alfalfa and red clover, the most important perennial legumes used as high-quality feed for livestock [21]. Equol derivatives have been detected extensively in ruminant milk [13,17,22,23] and are included in the Milk Composition Database [24]. Equol exhibits the highest estrogenic activity among all isoflavones and their metabolites [25]. Recent studies have shown that equol enhances osteoblastic bone formation, prevents bone loss, and protects cartilage and subchondral bone, demonstrating its bone-health properties [26,27]. Additionally, inverse relationships between equol production and various vascular pathologies, including cognitive impairment and dementia, have been observed [28].

*O*-desmethylangolensin (ODMA), another daidzein-derived gut microbiota catabolite, was also detected in milk in its conjugated form (Table 3), but it is not listed in the milk composition database. It was recently identified in cow’s milk by Roccheti et al. [1]. This is, however, the first time, to the best of our knowledge, that it has been found in goat’s milk. In addition, it is important to note that both equol and ODMA are not produced by all humans, as their occurrence is related to specific gut bacteria consortia [29,30].

Another interesting class of phenolic metabolites detected was phenyl-γ-valerolactones. These catabolites are produced by the gut microbiota through the metabolism of flavan-3-ols, and their production rate changes on the basis of the degree of polymerization, with monomers such as (epi)catechins being the main scaffolds for their formation [31,32]. Flavan-3-ols are widespread in forage and feeds [33,34], so their presence in animal milk is to be anticipated. Indeed, different types of phenyl-γ-valerolactones, including 5-(5′-hydroxyphenyl)-γ-valerolactone-3ʹ-glucuronide, have been reported previously in both raw and fermented milk [35]. The glucuronide was, however, not detected in the current study, where phenyl-γ-valerolactone sulfates predominated (Table 4). Phenyl-γ-valerolactones have been recently associated with a diversity of biological activities, including anti-inflammatory, neuroprotective, and cardioprotective effects [31,36]. There is a high inter-individual variation in the production of phenyl-γ-valerolactones [37,38] and these beneficial effects might depend on the capability of each individual to produce higher rather than lower amounts of these flavan-3-ol metabolites.

Other compounds typically found in crops and pastures are lignans, mainly represented by secoisolariciresinol and matairesinol [39]. Lignans are converted by gut microbiota into enterolactone and enterodiol, both of which may have estrogenic activity [39]. Both enterolignans were detected in milk samples, consistent with previous research [1,17,22,40]. Their production in humans is related to concentration gradients (low and high producers) and specific gut microbiota profiles [41], so the consumption of milk could be a strategy to reduce the inter-individual variability existing in their production in vivo. This is of interest since a recent prospective study has revealed that high plasma concentrations of enterolactone metabolites are linked to a lower risk of coronary artery disease [42]. They have also been inversely associated with type 2 diabetes mortality [43].

Urolithins, catabolites produced by the gut microbiota through the breakdown of ellagitannins and ellagic acid, may be related to the consumption of various herbs in ruminant feed [44]. In the current study, three urolithins, namely 3-hydroxy-urolithin, 3,8-dihydroxy-urolithin, and 3,8,9-trihydroxy-urolithin (Table 6), were identified in cow’s and goat’s milk. This is the first report on the presence of these microbial metabolites in animal milk. So far, the only information on urolithins in milk is related to human consumption and breast milk [45,46]. Since the gut microbiota plays a key role in urolithin formation, leading to a high individual variability, and some urolithins, in particular 3,8-dihydroxy-urolithin (also known as urolithin A) have been linked to several health benefits [47], the high amounts found in goat’s milk could be of interest when planning new nutritional strategies. In this sense, 3,8-dihydroxy-urolithin has been shown to improve mitochondrial function and muscle health, and it potentially also delays aging-related decline [48]. Moreover, 3-hydroxy-urolithin (urolithin B) has exhibited potential anti-obesity effects by modulating lipid metabolism [46].

Hippuric acid and hydroxybenzene derivatives were detected in all the milk samples, as reported in a specialized database [24]. These metabolites result from the metabolism of a wide variety of different phenolic compounds that occur in multiple dietary sources, including crops and forage [49,50]. They can also be produced endogenously from other substrates, including the aromatic amino acids phenylalanine and tyrosine [51].

The identification of different microbial metabolites in milk, such as equol, phenyl-γ-valerolactones, enterolactones, and urolithins, is of potential relevance, as they are not produced in vivo in broadly similar amounts by all individuals. Their production depends upon the presence and activity of specific gut microbiota bacteria/consortiums, which can vary among individuals [20,31,52,53]. In addition, many of the (poly)phenols from which these bioactive metabolites originate are present in plant-based foods such as soybean, pomegranate, and strawberry that are not consumed on a regular basis by much of the population. The consumption of milk, which is commonly consumed daily, might be a way of supplementing phenolic catabolites, in particular for individuals who cannot produce some of the individual metabolites. These insights may help to develop new dairy products rich in phenolic metabolites. In that case, studying the bioavailability of these milk metabolites will be relevant. The limited data that is currently available is promising, at least for equol [54] and urolithins [55,56].

The identification of these metabolites may have implications for the milk production chain. The use of (poly)phenol-rich feed for ruminants could be promoted, as some studies have shown that the concentration of these compounds in milk can be modulated by the diet [22,57,58,59]; an increase in the (poly)phenol content of the animal diet could lead to elevated levels of these colonic metabolites in milk-derived products, making them attractive to consumers seeking functional foods with potential health benefits. Nevertheless, other factors including the forage system, season, ruminal processes, post-ruminal changes, and animal health status may drive the amount of (poly)phenols in the milk and need to be considered [10,60]. Furthermore, some of the phenolic compounds identified serve as markers of various feeding regimes. For instance, in a recent study, the most discriminating compounds responsible for distinguishing milk samples based on animal feeding were hippuric acid, hydroxyhippuric acid, enterolactone, and isoflavone metabolites like equol and *O*-desmethylangolensin [61]. However, the use of hippuric acid in this context might be questionable in view of a recent publication by Clifford et al. [51]. In practice, determining the relationships among milk types, sterilization treatments, fat and lactose contents, and phenolic metabolite concentrations, is not straightforward. As noted above, metabolite concentrations are influenced by several factors [49,62], so a standardized approach starting from the same milk and applying various treatments will be required to better explore the influence of milk processing on the phenolic metabolite profile.

Regarding the comparison between animal species, it is remarkable that, except for lignan metabolites, goat’s milk exhibited a higher concentration of all the metabolites. Although limited research has been conducted comparing the content of these metabolites in cow’s and goat’s milk, similar findings were obtained for equol, which displayed a higher concentration in goat’s milk, and enterolactones, which exhibited a higher concentration in cow’s milk, in line with results of the current study [63]. These differences in metabolite profiles have been attributed to variations in the dietary supply and/or the microbial composition in the rumen, where the main bacterial communities in goat rumen are composed of Bacteroidetes, Firmicutes, and Proteobacteria, major families associated with equol producers [64,65].

## 4. Materials and Methods

### 4.1. Chemicals and Reagents

Analytical standards of daidzein-7-glucuronide, equol-7-glucuronide, equol-4ʹ-sulfate, and enterolactone were purchased from Toronto Research Chemicals (Toronto, ON, Canada). Hippuric acid was purchased from Sigma-Aldrich (St. Louis, MO, USA). 5-Phenyl-γ-valerolactone-3′-sulfate, 3-Hydroxy-urolithin-8-glucuronide, and Urolithin-3-glucuronide were prepared in-house using reported procedures [66]. HPLC-grade solvents (water, methanol, formic acid, and acetonitrile) and reagents were purchased from VWR International (Radnor, PA, USA).

### 4.2. Milk Samples

The milk samples used came from commercial products typically sold in Italy. They were purchased in a supermarket in Parma. Two different brands were used for each type of milk, except for the microfiltered cow’s and goat’s milk, where only one commercial milk was available (Table 1).

### 4.3. Sample Preparation

For sample preparation, 1 mL of each milk sample was frozen at −80 °C to aid protein precipitation. Once thawed, 25 μL of 35% phosphoric acid in H_2_O was added to denature and precipitate proteins. Samples were then vortexed and sonicated for 15 min at room temperature and centrifuged at 15,900× *g* for 15 min at 4 °C. After centrifugation, the supernatants were subjected to micro-elution solid-phase extraction (µSPE) [67]. Briefly, each sample (600 µL) was loaded onto a 96-well µSPE OASIS HLB plate (Eschborn, Germany), washed with water (200 µL) and 0.2% acetic acid (200 µL), and finally eluted with methanol (200 µL). All samples were stored at −80 °C prior to analysis.

### 4.4. Qualitative and Quantitative Analysis of Phenolic Compounds

Samples were analyzed through a UHPLC DIONEX Ultimate 3000 fitted with a TSQ Vantage triple-quadrupole mass spectrometer equipped with a heated-electrospray ionization (H-ESI-II) source (Thermo Fisher Scientifc Inc., San Jose, CA, USA). Chromatographic and ionization parameters were set following the method previously described by Brindani et al., with some modifications [66]. Briefly, separations were performed with a Kinetex EVO C18 (100 × 2.1 mm) of 2.6 µm particle size (Phenomenex, Torrance, CA, USA), with a pre-column (Phenomenex). For UHPLC, mobile phase A was 0.01% formic acid in water, and mobile phase B was acetonitrile containing 0.01% formic acid. The gradient started with 5% B, keeping isocratic conditions for 0.5 min, reaching 95% B at 7 min, followed by 1 min at 95% B and then 4 min under the starting conditions to re-equilibrate the column. The flow rate was set at 0.4 mL/min, the injection volume was 5 µL, and the column temperature was 40 °C. The MS worked in the negative ionization mode with the capillary temperature at 270 °C, while the source was at 300 °C. The sheath gas flow was 60 units, while the auxiliary gas pressure was set to 10 units. The source voltage was 3 kV. Ultra-high-purity argon gas was used for collision-induced dissociation (CID).

Up to 203 candidate phenolic metabolites were monitored in the selective reaction monitoring (SRM) mode. Two molecular transitions were used to qualify and quantify the phenolic compounds. Quantification was performed with calibration curves of standards when available, and when not, using the most structurally similar metabolite. Data processing was performed using Xcalibur software version 2.2.0.48 (Thermo Scientific Inc., Waltham, MA, USA). The nomenclature for phenolic metabolites follows the recommendations provided by Kay et al. [19].

### 4.5. Statistical Analysis

All samples were extracted in triplicate, and LC-MS analyses were performed for each sample extraction. Quantitative data are reported as mean ± standard deviation (SD). An analysis of variance (ANOVA) and Tukey’s multiple-range tests were carried out to compare milk samples. Statistical analysis was carried out using the IBM SPSS Statistics 23.0 software package (IBM, Chicago, IL, USA). The level of statistical significance was set at *p* < 0.05.

## 5. Conclusions

This study successfully identified 25 phenolic metabolites belonging to isoflavones, flavan-3-ols, lignans, ellagitannins, benzene diols, and hippuric acid families in commercial cow’s and goat’s milk. Ellagitannin-derived urolithins were detected for the first time in animal milk. Considering the high inter-individual variability in the production of most of the identified phenolic metabolites—some are produced only after the consumption of plant-based products that are not routinely consumed in some populations—the consumption of milk might be a strategy to increase the intake of bioactive phenolic metabolites. The supplementation of animals with (poly)phenol-rich diets may increase their metabolite concentrations in milk, allowing individuals who are unable to produce some of these metabolites to consume them through the daily intake of milk. In this manner, introducing goat’s milk into the diet may be a way to increase the intake of already-metabolized bioactive phenolic metabolites. But further studies are necessary to validate this statement and to estimate whether, despite the low concentrations in comparison with the (poly)phenols present in plant-based foods, milk-derived phenolics may have any effect on human health. Moreover, more research is needed to better explore the impact of animal feeds and milk processing on the final content of phenolic metabolites.

## Figures and Tables

**Table 1:** Codes for the samples included in the experimental design.

Milk Sample Type	Code *
Pasteurized whole cow’s milk	PWCa, PWCb
Semi-skimmed pasteurized cow’s milk	PSCa, PSCb
Whole UHT cow’s milk	UWCa, UWCb
Semi-skimmed UHT cow’s milk	USCa, USCb
Whole lactose-free UHT cow’s milk	ULCa, ULCb
Microfiltered semi-skimmed cow’s milk	MSC
Whole UHT goat’s milk	UWG
Semi-skimmed UHT goat’s milk	USG

* “a” and “b” represent two different brands of the same milk type.

**Table 2:** Qualitative analysis of (poly)phenolic metabolites detected in the milk samples.

Compound	Retention Time (min)	Parent Ion [M-H] (*m*/*z*)	S-lens	Quantifier Product Ion (*m*/*z*)	CE (V)	Qualifier Product Ion (*m*/*z*)	CE (V)
*Isoflavone metabolites*							
Dihydrogenistein-7-glucuronide	5.49	447	108	271	25	165	24
**Daidzein-7-glucuronide**	4.71	429	104	252	37	113	18
*O*-Desmethylangolensin-sulfate	7.63	337	136	257	20	108	34
Hydroxyequol-glucuronide	5.52	433	108	257	26	113	18
Hydroxyequol-sulfate	6.46	337	136	257	23		
**Equol-7-glucuronide**	5.27	417	108	113	22	241	26
Equol-7-sulfate	7.27	321	136	241	23	119	39
**Equol-4′-sulfate**	7.56	321	136	241	23	121	29
*Flavan-3-ol metabolites*							
5-(3′,4′,5′-Trihydroxyphenyl)-γ-valerolactone	5.54	223	75	205	12	138	26
5-(3ʹ/4ʹ-Hydroxyphenyl)-γ-valerolactone-sulfate	5.62	287	96	207	23	119	35
5-Phenyl-γ-valerolactone-4′-sulfate	5.04	271	93	191	23	147	35
**5-Phenyl-γ-valerolactone-3′-sulfate**	5.88	271	93	191	23	147	35
*Lignan metabolites*							
Enterolactone-glucuronide	5.49	473	108	297	25	113	20
Enterolactone-sulfate	7.89	377	136	297	25	253	26
**Enterolactone**	6.02	297	115	253	26	189	43
Enterodiol-sulfate	7.67	381	136	253	26		
*Ellagitannin metabolites*							
Dihydroxy-urolithin-glucuronide (urolithin C-glucuronide)	4.46	419	78	243	37	113	15
**3-Hydroxy-urolithin-8-glucuronide** **(also known as urolithin A-glucuronide)**	4.79	403	78	227	37	113	15
3-Hydroxy-urolithin-8-sulfate (urolithin A-sulfate)	6.67	307	78	227	19	183	19
**Urolithin-3-glucuronide** **(also known as urolithin B-glucuronide)**	5.37	387	83	211	35	113	17
Urolithin-3-sulfate (also known as urolithin B-sulfate)	8.23	291	78	211	19	167	19
*Hippuric acid derivatives*							
3′-Hydroxyhippuric acid	2.06	194	72	150	15	100	11
**Hippuric acid**	3.59	178	61	134	15	77.2	22
*Benzene diols derivatives*							
Benzene-1/2-ol-sulfate (isomer 1, catechol-sulfate 1)	2.22	189	70	109	20	81	20
Benzene-1/2-ol-sulfate (isomer 2, catechol-sulfate 2)	3.36	189	70	109	20	81	20

Compounds in bold were identified using authentic standards. Their nomenclature is according to Kay et al., 2020 (common names are reported in brackets) [19].

**Table 3:** Isoflavone metabolite composition of the milk samples.

	Compound * (ng/100 mL ± Standard Error)								
Milk Sample	DHG-7-G	D-7-G	ODMA-S	HE-G	HE-S	E-7-G	E-7-S	E-4-S	Total
PWCa	78 ± 13 ^d^	-	-	-	9.3 ± 3 ^c^	26± 5 ^f^	3.9 ± 0.2 ^c^	23 ± 5 ^d^	141 ± 6 ^c^
PWCb	89 ± 9 ^d^	19 ± 1 ^b^	4.5 ± 0.9 ^c^	-	4.6 ± 0.6 ^c^	84 ± 1 ^abc^	2.8 ± 0.1 ^c^	65 ± 9 ^cd^	269± 17 ^c^
PSCa	192 ± 49 ^bcd^	-	3.0 ± 0.1 ^c^	-	4.0 ± 0.9 ^c^	28 ± 8 ^ef^	6.3 ± 1.4 ^c^	46 ± 3 ^d^	280 ± 48 ^c^
PSCb	161 ± 43 ^cd^	34 ± 9 ^a^	4.8 ± 0.1 ^c^	7.8 ± 1.6 ^c^	3.8 ± 0.4 ^c^	118 ± 21 ^a^	12 ± 0.1 ^c^	59 ± 4 ^cd^	400 ± 67 ^c^
UWCa	142 ± 29 ^abc^	17 ± 3 ^b^	13 ± 1 ^c^	6.3 ± 1.0 ^c^	15 ± 1 ^c^	102 ± 12 ^ab^	19 ± 2 ^c^	163 ± 17 ^cd^	478 ± 33 ^c^
UWCb	229 ± 53 ^cd^	-	4.6 ± 0.2 ^c^	11 ± 0.5 ^c^	23 ± 4 ^c^	42 ± 3 ^def^	10 ± 3 ^c^	253 ± 9 ^c^	591 ± 1 ^c^
USCa	159 ± 36 ^cd^	34 ± 9 ^a^	14 ± 1 ^c^	17 ± 2 ^c^	23 ± 2 ^c^	109 ± 22 ^ab^	17 ± 3 ^c^	136 ± 4 ^cd^	501 ± 20 ^c^
USCb	140 ± 35 ^cd^	15 ± 2 ^b^	5.7 ± 0.2 ^c^	9.4 ± 0.3 ^c^	6.4 ± 1.5 ^c^	84 ± 13 ^abc^	11 ± 3 ^c^	91 ± 19 ^cd^	353 ± 10 ^c^
ULCa	307 ± 50 ^a^	-	17 ± 1 ^c^	11 ± 1 ^c^	13 ± 3 ^c^	64 ± 8 ^cde^	36 ± 9 ^c^	159 ± 25 ^cd^	607 ± 81 ^c^
ULCb	280 ± 52 ^ab^	-	13 ± 4 ^c^	18 ± 2 ^c^	8.1 ± 0.1 ^c^	74 ± 5 ^bcd^	25 ± 5 ^c^	172 ± 40 ^cd^	590 ± 92 ^c^
MSC	179 ± 2 ^bcd^	-	10 ± 2 ^c^	4.2 ± 0.3 ^c^	15 ± 4 ^c^	81 ± 14 ^bc^	7 ± 2 ^c^	123 ± 27 ^d^	419 ± 46 ^c^
UWG	280 ± 48 ^ab^	-	101 ± 10 ^b^	131 ± 12 ^b^	278 ± 3 ^b^	87 ± 17 ^abc^	457 ± 88 ^b^	1016 ± 207 ^b^	2350 ± 374 ^b^
USG	143 ± 35 ^cd^	17 ±1 ^b^	313 ± 1 ^a^	188 ± 38 ^a^	616 ± 78 ^a^	43 ± 3 ^def^	1044 ± 120 ^a^	1540 ± 108 ^a^	3904 ± 345 ^a^

* DHG-7-G, Dihydrogenistein-7-glucuronide; D-7-G, Daidzein-7-glucuronide; ODMA-S, O-Desmethylangolensin-sulfate; HE-G, Hydroxyequol-glucuronide; HE-S, Hydroxyequol-sulfate; E-7-G, Equol-7-glucuronide; E-7-S, Equol-7-sulfate; E-4-S, Equol-4′-sulfate. Different superscript letters indicate statistically significant differences among samples.

**Table 4:** Flavan-3-ol metabolite composition of the milk samples.

	Compound * (ng/100 mL ± Standard Error)				
Milk Sample	THP-γ-VL	HP-γ-VL-S	P-γ-VL-4-S	P-γ-VL-3-S	TOTAL
PWCa	140 ± 18 ^a^	41 ± 4 ^c^	56 ± 3 ^d^	65 ± 11 ^c^	303 ± 16 ^c^
PWCb	111 ± 11 ^a^	31 ± 1 ^c^	131 ± 20 ^c^	95 ± 7 ^c^	367 ± 30 ^c^
PSCa	30 ± 2 ^cd^	28 ± 5 ^c^	46 ± 12 ^d^	56 ± 1 ^c^	161 ± 10 ^c^
PSCb	56 ± 1 ^bc^	45 ± 8 ^c^	44 ± 11 ^d^	96 ± 11 ^c^	241 ± 10 ^c^
UWCa	71 ± 12 ^b^	77 ± 15 ^c^	38 ± 5 ^d^	133 ± 11 ^c^	320 ± 31 ^c^
UWCb	45 ± 13 ^bcd^	60 ± 16 ^c^	44 ± 10 ^d^	38 ± 11 ^c^	188 ± 7 ^c^
USCa	71 ± 15 ^b^	96 ± 10 ^c^	41 ± 5 ^d^	122 ± 13 ^c^	330 ± 5 ^c^
USCb	20 ± 2 ^d^	42 ± 4 ^c^	36 ± 5 ^d^	75 ± 16 ^c^	173 ± 20 ^c^
ULCa	64 ± 6 ^b^	61 ± 10 ^c^	47 ± 14 ^d^	601 ± 14 ^c^	233 ± 43 ^c^
ULCb	-	63 ± 6 ^c^	50 ± 8 ^d^	57 ± 9 ^c^	170 ± 13 ^c^
MSC	76 ± 13 ^b^	72 ± 2 ^c^	23 ± 5 ^d^	80 ± 8 ^c^	252 ± 17 ^c^
UWG	-	333 ± 54 ^b^	216 ± 33 ^b^	1161 ± 79 ^b^	1710 ± 165 ^b^
USG	-	2676 ± 122 ^a^	497 ± 29 ^a^	14,101 ± 692 ^a^	17,274 ± 827 ^a^

* THP-γ-VL, 5-(3′,4′,5′-Trihydroxyphenyl)-γ-valerolactone; HP-γ-VL-S, 5-(Hydroxyphenyl)-γ-valerolactone-sulfate; P-γ-VL-4-S, 5-Phenyl-γ-valerolactone-4′-sulfate; P-γ-VL-3-S, 5-Phenyl-γ-valerolactone-3ʹ-sulfate. Different superscript letters indicate statistically significant differences among samples.

**Table 5:** Lignan metabolite composition of the milk samples.

Compound * (ng/100 mL ± Standard Error)				
Milk Sample	EL-G	EL-S	EL	TOTAL
PWCa	779 ± 26 ^a^	116 ± 26 ^c^	97 ± 11 ^ef^	992 ± 199 ^bcd^
PWCb	698 ± 182 ^a^	84 ± 1 ^c^	66 ± 22 ^ef^	848 ± 201 ^cde^
PSCa	744 ± 24 ^a^	189 ± 2 ^c^	56 ± 1 ^f^	990 ± 22 ^bcd^
PSCb	770 ± 98 ^a^	177 ± 14 ^c^	110 ± 17 ^ef^	1057 ± 113 ^bcd^
UWCa	780 ± 162 ^a^	538 ± 62 ^a^	188 ± 10 ^cde^	150 ± 187 ^a^
UWCb	756 ± 167 ^a^	427 ± 89 ^ab^	152 ± 5 ^cdef^	1335 ± 183 ^ab^
USCa	65 ± 15 ^b^	602 ± 110 ^a^	14 ± 42 ^def^	810 ± 73 ^def^
USCb	57 ± 5 ^b^	256 ± 19 ^bc^	124 ± 36 ^def^	437 ± 33 ^fg^
ULCa	125 ± 21 ^b^	579 ± 83 ^a^	278 ± 62 ^c^	982 ± 112 ^bcd^
ULCb	158 ± 37 ^b^	55 ± 104 ^a^	833 ± 3 ^a^	1496 ± 102 ^a^
MSC	72 ±2 ^b^	201 ± 64 ^c^	121 ± 24 ^def^	394 ±50 ^g^
UWG	113 ± 17 ^b^	181 ± 37 ^c^	242 ± 23 ^cd^	535± 42 ^efg^
USG	88 ± 17 ^b^	439 ± 31 ^a^	667 ± 109 ^b^	119 ± 125 ^abc^

* THP-γ-VL, 5-(3′,4′,5′-Trihydroxyphenyl)-γ-valerolactone; HP-γ-VL-S, 5-(Hydroxyphenyl)-γ-valerolactone-sulfate; P-γ-VL-4-S, 5-Phenyl-γ-valerolactone-4′-sulfate; P-γ-VL-3-S, 5-Phenyl-γ-valerolactone-3′-sulfate. Different superscript letters indicate statistically significant differences among samples.

**Table 6:** Urolithins in the milk samples.

Compound * (ng/100 mL ± Standard Error)						
Milk Sample	DHU-G	3-HU-8-G	3-HU-8-S	U-3-G	U-3-S	TOTAL
PWCa	427 ± 29 ^a^	33 ± 7 ^bc^	4.8 ± 1.5 ^c^	-	-	465 ± 32 ^c^
PWCb	237 ± 27 ^cd^	37 ± 3 ^b^	-	-	-	274 ± 28 ^c^
PSCa	256 ± 27 ^bcd^	43 ± 9 ^b^	8.3 ± 1.6 ^c^	-	12 ± 2 ^c^	319 ± 38 ^c^
PSCb	416 ± 17 ^ab^	65 ± 11 ^a^	9.6 ± 1.8 ^c^	-	28 ± 1 ^c^	514 ± 26 ^c^
UWCa	266 ± 46 ^bcd^	39 ± 9 ^b^	16 ± 5 ^c^	-	8.4 ± 0.1 ^c^	329 ± 57 ^c^
UWCb	289 ± 68 ^abcd^	34 ± 9 ^bc^	36 ± 7 ^c^	-	6.5 ± 1.9 ^c^	365 ± 61 ^c^
USCa	224 ± 58 ^cde^	12 ± 1 ^d^	25 ± 1 ^c^	-	96 ± 14 ^c^	357 ± 70 ^c^
USCb	365 ± 18 ^abc^	41 ± 7 ^b^	28 ± 8 ^c^	-	43 ± 4 ^c^	476 ± 25 ^c^
ULCa	244 ± 48 ^cd^	7 ±1 ^cd^	10 ± 1 ^c^	-	30 ± 7 ^c^	301 ± 54 ^c^
ULCb	406 ± 13 ^ab^	5.7 ± 0.6 ^d^	6.6 ± 1 ^c^	-	4.2 ± 0.4 ^c^	423 ± 131 ^c^
MSC	264 ± 67 ^bcd^	33 ± 2 ^bc^	5.7 ± 1.4 ^c^	-	8.8 ± 1.1 ^c^	311 ± 69 ^c^
UWG	145 ± 4 ^de^	-	2414 ± 262 ^b^	222 ± 59 ^b^	2652 ± 334 ^b^	5433 ± 605 ^b^
USG	76 ± 10 ^e^	-	81534 ± 17 ^a^	2647 ± 455 ^a^	37,581 ± 1956 ^a^	121,839 ± 2104 ^a^

* DHU-G, Dihydroxy-urolithin-glucuronide; 3-HU-8-G, 3-Hydroxy-urolithin-8-glucuronide; 3-HU-8-S, 3-Hydroxy-urolithin-8-sulfate; U-3-G, Urolithin-3-glucuronide; U-3-S, Urolithin-3-sulfate. Different superscript letters indicate statistically significant differences among samples.

**Table 7:** Hippuric acids in the milk samples.

Compound * (µg/100 mL ± Standard Error)			
Milk Sample	3′-HHA	HA	TOTAL
PWCa	25 ± 5 ^c^	1847 ± 226 ^ab^	1872 ± 228 ^b^
PWCb	10 ± 2 ^c^	1381 ± 313 ^bc^	1391 ± 312 ^bc^
PSCa	13 ± 2 ^c^	1215 ± 172 ^c^	1228 ± 174 ^c^
PSCb	16 ± 1 ^c^	1245 ± 21 ^c^	1261 ± 21 ^c^
UWCa	17 ± 2 ^c^	12401 ± 82 ^c^	1258 ± 84 ^c^
UWCb	18 ± 4 ^c^	1495 ± 171 ^bc^	1514 ± 175 ^bc^
USCa	15 ± 1 ^c^	1197 ± 86 ^c^	1211 ± 89 ^c^
USCb	19 ± 4 ^c^	1378 ± 234 ^bc^	1397 ± 23 ^bc^
ULCa	14 ± 1 ^c^	1349 ± 133 ^bc^	1363 ± 134 ^bc^
ULCb	11 ± 2 ^c^	1427 ± 283 ^bc^	1438 ± 284 ^bc^
MSC	16 ± 3 ^c^	1306 ± 120 ^bc^	1322 ± 122 ^bc^
UWG	67 ± 13 ^b^	1435 ± 148 ^bc^	1502 ± 158 ^bc^
USG	169 ± 15 ^a^	2366 ± 218 ^a^	2535 ± 228 ^a^

* 3-HHA, 3′-Hydroxyhippuric acid; HA, Hippuric acid. Different superscript letters indicate statistically significant differences among samples.

**Table 8:** Benzene diol composition of the milk samples.

Compound * (ng/100 mL ± Standard Error)			
Milk Sample	B-S1	B-S2	TOTAL
PWCa	5.4± 0.5 ^c^	4.3 ± 0.2 ^c^	9.7 ± 0.6 ^c^
PWCb	6.5 ± 0.6 ^bc^	2.8 ± 0.8 ^c^	9.3 ± 0.4 ^c^
PSCa	2.3 ± 0.4 ^c^	1.7 ± 0.1 ^c^	4.0 ± 0.4 ^c^
PSCb	2.8 ± 0.3 ^c^	4.6 ± 1.3 ^c^	7.4 ± 1.5 ^c^
UWCa	3.8 ± 0.6 ^c^	3.4 ± 0.8 ^c^	7.2 ±1.4 ^c^
UWCb	3.9 ± 0.9 ^c^	4.3 ± 1.1 ^c^	8.2 ± 1.8 ^c^
USCa	2.8 ± 0.6 ^c^	3.6 ± 0.6 ^c^	6.4 ± 0.6 ^c^
USCb	3.8 ± 0.4 ^c^	2.2 ± 0.4 ^c^	6.0 ± 0.4 ^c^
ULCa	4.0 ± 0.4 ^c^	2.8 ± 0.1 ^c^	4.8 ± 0.4 ^c^
ULCb	56 ± 0.5 ^c^	2.7 ± 0.6 ^c^	8.3 ± 1.0 ^c^
MSC	3.7 ± 0.6 ^c^	4.5 ± 0.8 ^c^	8.1 ± 1.4 ^c^
UWG	11 ± 1 ^b^	30 ± 6 ^b^	41 ± 5 ^b^
USG	98 ± 5 ^a^	45 ± 4 ^a^	143 ± 9 ^a^

* B-S1, Hydroxybenzene-sulfate (isomer 1); B-S2, Hydroxybenzene-sulfate (isomer 2). Different superscript letters indicate statistically significant differences among samples.

## Data Availability

Data are contained within the article.
